# A Pyrimidin-Like Plant Activator Stimulates Plant Disease Resistance and Promotes the Synthesis of Primary Metabolites

**DOI:** 10.3390/ijms21082705

**Published:** 2020-04-14

**Authors:** Jian Li, Ting Long, Tie-Jun Sun, Yun Lu, Jian Yin, Yu-Bing Yang, Guang-Yi Dai, Xiao-Yuan Zhu, Nan Yao

**Affiliations:** 1 State Key Laboratory of Biocontrol, Guangdong Provincial Key Laboratory of Plant Resources, School of Life Sciences, Sun Yat-sen University, Guangzhou 510275, China; lijian76@mail.sysu.edu.cn (J.L.); longt_2020@163.com (T.L.); shida_tiejun@163.com (T.-J.S.); luyungg@163.com (Y.L.); yinjian6@mail.sysu.edu.cn (J.Y.);; 2 Plant Protection Research Institute, Guangdong Academy of Agricultural Sciences, Guangzhou 510640, China; zhuxy@gdppri.com

**Keywords:** plant activator, plant resistance, jasmonic acid, primary metabolites, rice

## Abstract

Plant activators are chemicals that induce plant defense responses to various pathogens. Here, we reported a new potential plant activator, 6-(methoxymethyl)-2-[5-(trifluoromethyl)-2-pyridyl] pyrimidin-4-ol, named PPA2 (pyrimidin-type plant activator 2). Unlike the traditional commercial plant activator benzothiadiazole *S*-methyl ester (BTH), PPA2 was fully soluble in water, and it did not inhibit plant growth or root system development in rice (*Oryza sativa*). PPA2 pretreatment significantly increased plant resistance against bacterial infection in both *Arabidopsis* and rice, in conjunction with increases in the level of jasmonoyl-isoleucine and 12-oxo-phytodienoic acid. In addition, metabolite profiling indicated that BTH significantly reduced the abundance of various primary metabolites in rice seedlings, including most amino acids, sugars, and organic acids; by contrast, PPA2 promoted their synthesis. Our results thus indicate that PPA2 enhances plant defenses against bacterial infection through the jasmonic acid pathway, and that as a water-soluble compound that can promote the synthesis of primary metabolites it has broad potential applications in agriculture.

## 1. Introduction

In nature, plants are continuously subjected to attack by microbial pathogens and herbivorous insects. To combat infection by these deleterious organisms, plants often switch to a primed state of enhanced defense [1]. Plant activators are natural or synthetic chemical compounds that protect plants from various pathogens by activating the defense priming [2]. Plant activators provide durable effects to enhance plant resistance, unlike traditional fungicides or pesticides since they do not exert a direct detrimental effect on the pathogens [3]. The application of plant activators in crops is on the rise to control currently emerging plant diseases [4]; however, most of the molecular mechanisms underlying immune induction by these activators remain unclear.

In recent years, some plant activators have been successfully developed and applied in agriculture, such as 2,6-dichloroisonicotinic acid and its methyl ester (both referred to as INA), benzo(1,2,3)thiadiazole-7-carbothioic acid *S*-methyl ester (BTH), and non-protein amino acid β-aminobutyric acid (BABA) [2]. The majority of plant activators activate defense responses that resemble systemic acquired resistance (SAR) or induced systemic resistance [5]. For example, BTH, a traditional plant activator originally developed as a synthetic analog of the plant hormone salicylic acid (SA), can provoke plant disease-resistance responses by stimulating SAR [6]. It has been shown that BTH regulates *Arabidopsis* SAR and defense priming by the downstream action of accumulation of mRNA transcripts and inactive proteins of mitogen-activated protein kinase 3 (MPK3) and MPK6 [7], indicating that SA-dependent immunity plays an important role in plant activator-induced defense priming. In *Arabidopsis thaliana*, BTH treatment restricts the growth of the pathogen *Pseudomonas syringae* stain DG3 [8]. It has been also reported that BTH induces resistance to the bacterial pathogen *Xanthomonas oryzae* pv. *oryzae* in rice [9]. Besides SA, the plant hormone jasmonic acid (JA) also plays a central role in manipulating defense priming. Jasmonoyl-isoleucine (JA-Ile) and 12-oxo-phytodienoic acid (OPDA) could be induced in hexanoic acid-primed tomato plants after *Botrytis cinerea* inoculation [10]. Treatment with 3-pentanol significantly reduced the disease severity caused by *Xanthomonas axonopodis* pv*. vesicatoria* and the naturally occurring *Cucumber mosaic virus* by priming JA signaling in pepper [11]. Up to now, the agricultural applications of plant activators have remained far from developed, and most of the compounds (including BTH) cannot dissolve in water; thus, they may also cause secondary pollution from the organic solvent.

Primary metabolites, such as amino acids, carbohydrates, and organic acids, play a major role in plant cell-maintenance, development, and reproduction [12]. Amino acids participate in a variety of metabolic processes and are necessary for the transportation and storage of all nutrients, including carbohydrates, proteins, vitamins, and minerals [13]. Sugars are ubiquitous and critical components of the general metabolism that are also primary products of photosynthesis, and thus have critical effects on plant growth and development [14]. Organic acids, resulting from the incomplete oxidation of photosynthetic products, have a vital role in the conversion of carbon compounds into metabolic pathways [15]. Since there is a refined balanced between plant growth and defense, whether plant activators can improve plant resistance through regulating the primary metabolite metabolism remains unclear.

In this study, we reported a new pyrimidine-type plant activator, 6-(methoxymethyl)-2-[5-(trifluoromethyl)-2-pyridyl] pyrimidin-4-ol, named as pyrimidin-type plant activator 2 (PPA2). We investigated its effects on defense responses in *Arabidopsis* and rice. Unlike BTH, PPA2 was a water-soluble compound and did not inhibit plant growth or root development. Nonetheless, we found that PPA2 can induce immune responses against bacterial infection in both *Arabidopsis* and rice, the mechanism of which might be related to the reactive oxygen species (ROS) and JA pathway. Moreover, whereas BTH reduces the abundance of most primary metabolites, including amino acids, sugars, and organic acids, PPA2 promotes the synthesis of certain types of primary metabolites. Therefore, PPA2 may have broad potential applications in agriculture.

## 2. Results

### 2.1. PPA2 Stimulates Arabidopsis Resistance to Bacterial Infection

**Figure 1:**
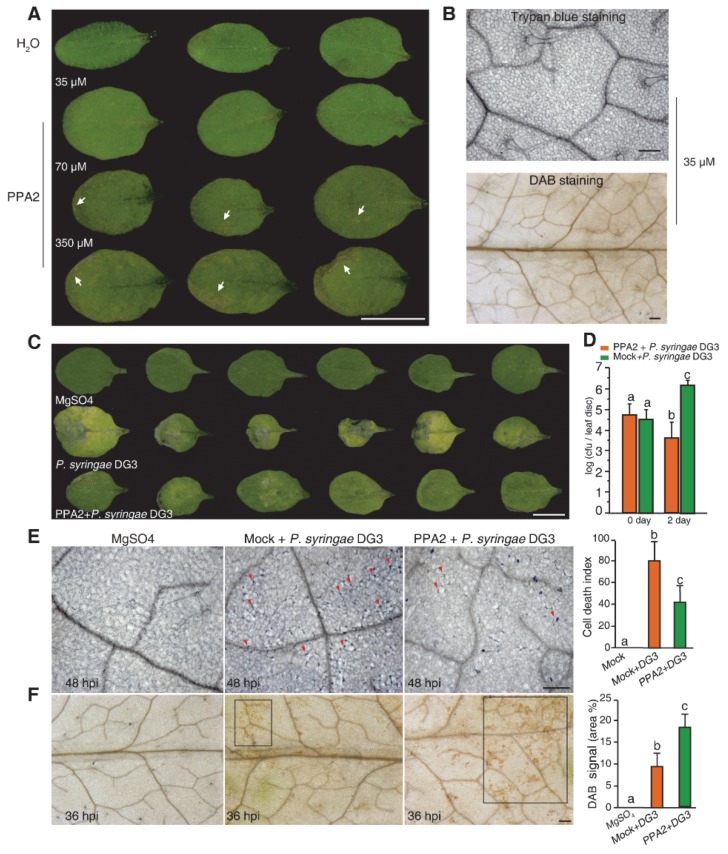
PPA2 can enhance plant resistance to the *P. syringae* virulent strain DG3. (**A**) PPA2 did not harm *Arabidopsis* leaves under 35 μM. Plants were sprayed with different concentrations of PPA2 (dissolved in distilled water) and were observed eight days later. Note brown spots (white arrows) in 70 μM and 350 μM PPA2 treated leaves. Bar = 10 mm. (**B**) Trypan blue and 3,3′-diaminobenzidine tetrahydrochloride (DAB) staining were used for detecting cell death and ROS production after 35 μM PPA2 treatments for 3 days. Bars = 200 μm. (**C**) PPA2 pretreatment inhibited cell death caused by bacterial infection. Cell death symptoms in plants pretreated with 35 μM PPA2 for three days, followed by infiltration of 10 mM MgSO_4_ (Mock) or the *Pseudomonas syringae* pv. *maculicola* strain DG3 (OD_600_ = 0.005) three days post-inoculation. Bar = 10 mm. (**D**) Growth of the *P. syringae* virulent strain DG3 in wild-type plants with or without 35 μM PPA2 pretreatment after infection at OD_600_ = 0.001. Lesion-free plants were inoculated with bacteria at three weeks of age. Different letters indicate significant differences at *P* < 0.05 as assessed using a post hoc multiple *t*-test. This experiment was repeated at least three times using independent samples. Error bars represent the means ± SE from six replicates in each experiment. (**E**) Images of cell death in H_2_O- and PPA2-pretreated plants after inoculation with 10 mM MgSO_4_ (Mock) or the *P. syringae* virulent strain DG3 (DG3). Dead cells were detected by trypan blue staining at 48 hpi. Note dead cells (red arrowheads) in the inoculated leaves. A statistical analysis of the degree of cell death (the cell death index) in each group was shown at the right side. The values are the means ± SE of at least three independent replicates. Scale bar = 200 μm. (**F**) Images of H_2_O_2_ signals in H_2_O- and PPA2-pretreated plants after inoculation with the *P. syringae* virulent strain DG3. The H_2_O_2_ signals were detected by DAB staining at 36 hpi. Note the brown DAB deposits (black squares). A statistical analysis of the H_2_O_2_ signal in each group was shown at the right side. The values are the means ± SE of at least three independent replicates. Scale bar = 200 μm.

Plant activators include natural or synthetic compounds. Previously, we found a molecule, 6-(methoxymethyl)-2-[5-(trifluoromethyl)-2-pyridyl] pyrimidin-4-ol, and its chemical structure has a similarity to the previously reported plant activator PPA [8]. Therefore, we named this synthetic compound PPA2 (pyrimidin-type plant activator 2). To test whether PPA2 has a plant activator function to stimulate plant defense responses, we first examined the effect of a suitable concentration of PPA2 on the plant phenotype. After spraying plants with different concentrations of PPA2, we found that 35 μM PPA2 had no harmful effects on the plants, whereas 350 μM PPA2, a standard concentration of the well-known plant activator BTH [16], induced visible cell death (Figure 1A). We further confirmed no macroscopic cell death or H_2_O_2_ production in 35 μM PPA2-treated leaves identified by trypan blue staining and 3,3′-diaminobenzidine tetrahydrochloride (DAB) staining (Figure 1B). Therefore, we used this concentration for further studies on *Arabidopsis*.

To determine whether PPA2 pretreatment could increase plant resistance, we applied 35 μM PPA2 to plants for three days and then inoculated them with the bacterial pathogen *P. syringae* DG3. At three days post-inoculation (dpi), PPA2-pretreated leaves demonstrated dramatically reduced disease symptoms compared to the infected leaves without PPA2 (Figure 1C), as well as significantly less bacterial growth (Figure 1D). In addition, trypan blue staining indicated that the PPA2-pretreated leaves had fewer dead cells at 48 h post-inoculation (hpi; Figure 1E). Moreover, at 36 hpi, PPA2-pretreated leaves showed significantly increased DAB deposits compared with the bacterial infected leaves without PPA2 (Figure 1F), demonstrating that PPA2-pretreated plants infected with bacteria experienced a stronger and earlier H_2_O_2_ burst than did the mock-treated plants. Together, these results indicated that PPA2 effectively protected plant cells against bacterial infection through regulating ROS production.

To investigate whether PPA2 could also boost plant defenses against the pathogenic fungus *Botrytis cinerea*, we first tested the effect of PPA2 on spore germination to demonstrate whether PPA2 directly affected the spores. The percentage of spores that had germinated at 12 hpi showed no difference between the PPA2-treated group and mock (water-treated) groups (Figure S1). Furthermore, three-week-old *Arabidopsis* were sprayed with 35 μM PPA2 or water for three days and were then inoculated with *B. cinerea* spores. After five days post the infection, we observed no difference in disease symptoms with or without PPA2 pretreatment (Figure S2A), and the same applied to the ROS burst and cell death detections (Figure S2B,C). The results indicated that PPA2 did not change the resistance of *Arabidopsis* plants to *B. cinerea*.

### 2.2. PPA2 Stimulates Rice Resistance to Xanthomonas Oryzae Infection

**Figure 2:**
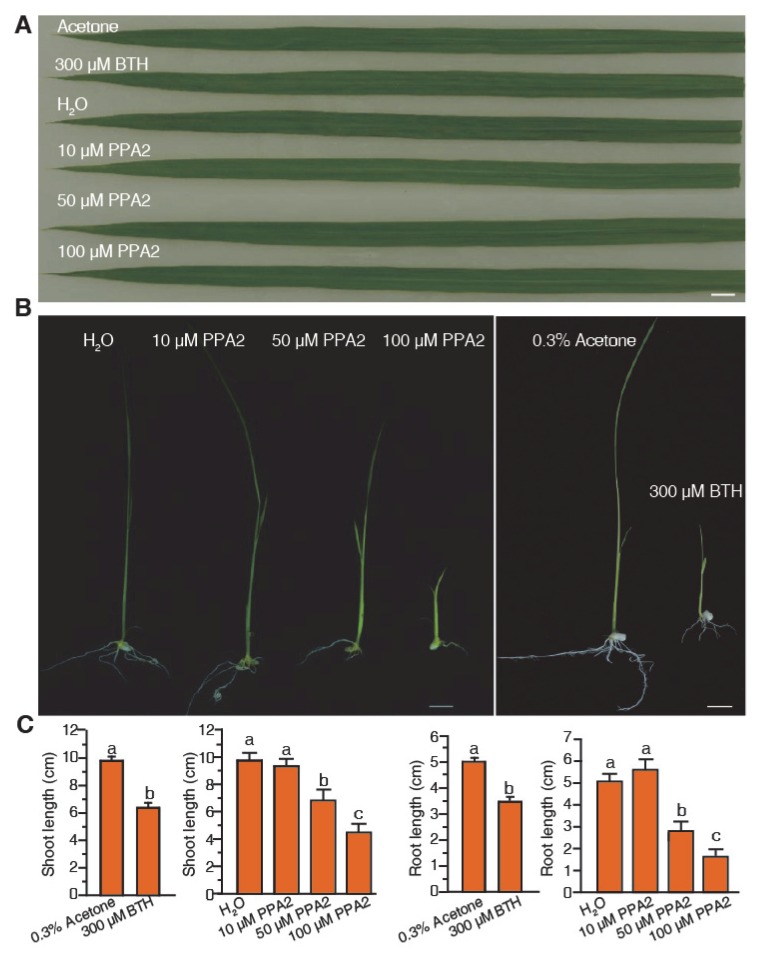
PPA2 does not affect the normal growth of rice at 10 μM. (**A**) The leaf phenotype of rice pretreated with various concentrations of PPA2. Five-six-leaf stage rice seedlings were sprayed with BTH and three different concentrations of PPA2, respectively. The image was recorded after treatment for five days. Bar = 1 cm. (**B**,**C**) The effect of PPA2 on the rice seedling growth. The seedlings were cultivated on 0.15% agar medium containing various reagents (H_2_O, 10 μM PPA2, 50 μM PPA2, 100 μM PPA2, 0.3% acetone, or 300 μM BTH). (**B**) Photos were taken at 14 days after the start of treatment. (**C**) Measurement of shoot height and root length of rice seedlings. Different letters above bars indicate significant differences as assessed by a post hoc multiple *t*-test (*p* < 0.05). This experiment was repeated twice by using independent samples.

In order to study whether PPA2 affects rice resistance, we first sprayed five-six-leaf stage rice seedlings with three different concentrations of PPA2 for five days to determine a suitable concentration. We found that no cell death phenotypes were observed after three different concentrations of PPA2 treatment in rice leaves (Figure 2A). We also explored whether PPA2 affected rice growth by analyzing the phenotype of rice seedlings cultivated for 14 days on 0.15% agar medium containing either PPA2 or BTH. We found that neither the height nor root length of rice seedlings showed any difference after 10 μM PPA2 treatment when compared with the water-treated group. However, the PPA2 treatment above a 50 μM concentration seriously affected the rice growth (Figure 2B). The shoot height and root length were significantly inhibited after 100 μM PPA2 and 300 μM BTH treatments (Figure 2B,C). We used 10 μM PPA2 for further studies in rice.

**Figure 3:**
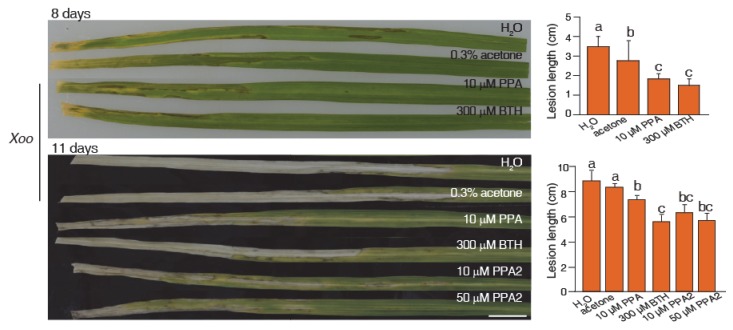
PPA2 can enhance the resistance of rice to *Xanthomonas oryzae.* Rice leaves pretreated with 0.3% acetone (solvent of BTH), 300 μM BTH, and 10 μM PPA or PPA2 for three days and then inoculated with *Xoo*. The phenotype was recorded after treatment for 8 and 11 days. Bar = 10 mm. Statistical analysis of lesion lengths in different groups. Different letters above bars indicated significant differences as assessed by a post hoc multiple *t*-test (*p* < 0.05). The experiment was repeated twice by using independent samples.

To investigate plant resistance induced by PPA2 in rice, we used *Xanthomonas oryzae* pv. *oryzae* (*Xoo*), the causal pathogen of rice blight, to confirm whether PPA2 had antibacterial activity in vitro. As shown in Figure S3A, by using a turbidimeter test, we found that PPA2 did not inhibit *Xoo* growth in vitro, reflecting a characteristic feature of plant activators similar to BTH. Then, we tested whether PPA2 could enhance rice resistance to *Xoo*. We sprayed the rice seedlings with 0.3% acetone (solvent of BTH), 300 μM BTH, 10 μM PPA, or 10 μM PPA2. Three days later, we used the leaf-clipping method to inoculate leaves with *Xoo* (GD-IV strain). We recorded and analyzed the lengths of leaf lesions. As shown in Figure 3 and Figure S3B, the lesion lengths in the 300 μM BTH-pretreatment and 10 μM PPA or PPA2-pretreatment groups were significantly shorter than those in the control group, indicating that PPA2 can activate resistance against *Xoo* infection and exhibited a similar function to previously reported plant activators (BTH and PPA).

We also explored whether PPA2 could enhance rice defense against *Magnaporthe grisea*, a fungus that infects rice. We pretreated rice seedlings with different concentrations of PPA2 for 3 days, then inoculated them with the pathogen, and the disease lesions were recorded 7 dpi (Figure S4A). We found that BTH pretreatment contributed significantly to rice defense against *M. grisea*, which is consistent with what was previously report [17], whereas PPA2 pretreatment failed to stop rice blast infection (Figure S4B).

### 2.3. Hormones Accumulate in PPA2-Pretreated Rice in Response to Xoo Infection

**Figure 4:**
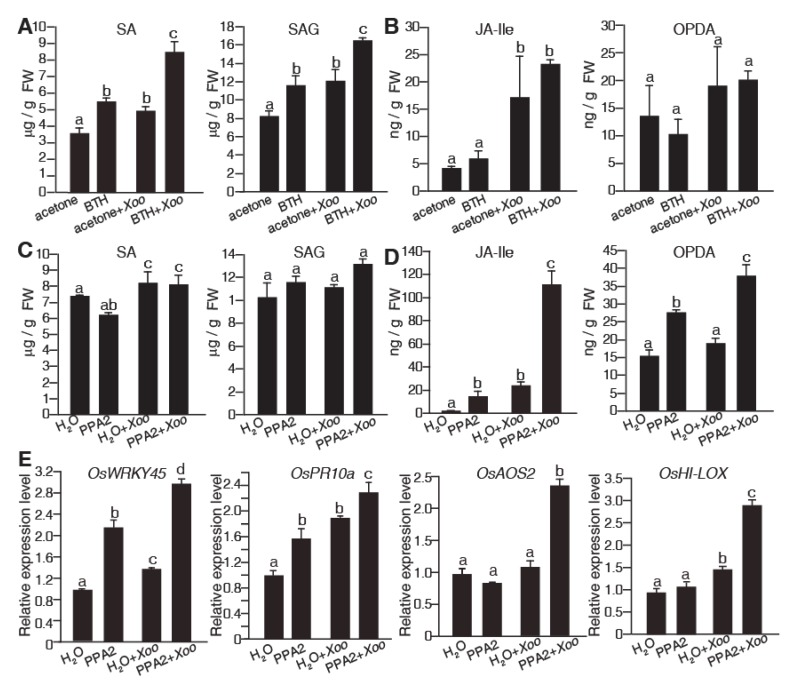
Influence of PPA2 on hormone accumulation in rice. (**A−D**) Five-six-leaf stage rice plants were sprayed with 300 μM BTH or 10 μM PPA2 for three days and then inoculated with *Xoo* for five days. Plant hormones were extracted following the steps described in Methods and identified by HPLC-ESI-MS. The levels of SA, SAG, JA-Ile, and OPDA after BTH plus *Xoo* treatments (**A**,**B**) or PPA2 plus *Xoo* treatments (**C**,**D**) were quantified. The experiment was repeated three times by using independent samples. Different letters represent a significant difference as assessed by using a post hoc multiple *t*-test (*p* < 0.05). FW, fresh weight. (**E**) The relative transcript level of *WRKY45* (Os*05g0322900*), *PR10a* (Os*12g0555500*), *AOS2* (Os*03g0225900*), and *HI-LOX* (*Os08g39840*). After the water or 10 μM PPA2 treatments for three days, rice leaves were inoculated with *Xoo*. The RNA samples were collected 24 h post inoculation. *UBQ* (Os*03g0234200*) transcript levels were used as the internal control. Gene expression values are presented relative to the values for the group treated with H_2_O (set as 1). Samples were analyzed in triplicate by using independent samples. Different letters represent significant differences as assessed by using a post hoc multiple *t*-test (*p* < 0.05).

Plant resistance often involves in hormonal signaling pathways. To identify which hormone played a role in the defense response of PPA2-treated rice plants, we measured the contents of hormones including salicylic acid (SA), salicylic acid 2-O-β-D-glucose (SAG), jasmonoyl-isoleucine (JA-Ile), and 12-oxo-phytodienoic acid (OPDA) in rice. As shown in Figure 4A, both SA and SAG accumulated in rice plants after 300 μM BTH treatment for three days when compared to the acetone control treatment. Furthermore, as supported by previous phenotypic observations, pretreatment with BTH significantly increased both levels of SA and SAG after a challenge with the bacterial pathogen *Xoo*. However, we found that no significant change of the JA-Ile and OPDA levels were detected with BTH or without BTH pretreatments in response to a pathogen attack (Figure 4B). In contrast, the content of JA-Ile and OPDA greatly increased in rice plants both pretreated alone with PPA2 for three days and inoculated with *Xoo* after PPA2 pretreatments (Figure 4D). No significant changes of SA and SAG levels were measured after PPA2 treatments (Figure 4C), indicating that the SA pathway was not involved in PPA2-induced defense responses. We also detected the transcript levels of resistance genes in plants pretreated with PPA2 for three days and then inoculated with *Xoo* for 24 h. The results showed that the JA-related genes (*OsWRKY45*, *OsPR10a*, *OsAOS2,* and *OsHI-LOX*) [18] were significantly up-regulated in PPA2-treated plants (Figure 4E). Taken together, these results indicated that PPA2 may enhance plant resistance by inducing the JA pathway.

### 2.4. PPA2 Induces Accumulation of Certain Primary Metabolite Contents in Rice

**Figure 5:**
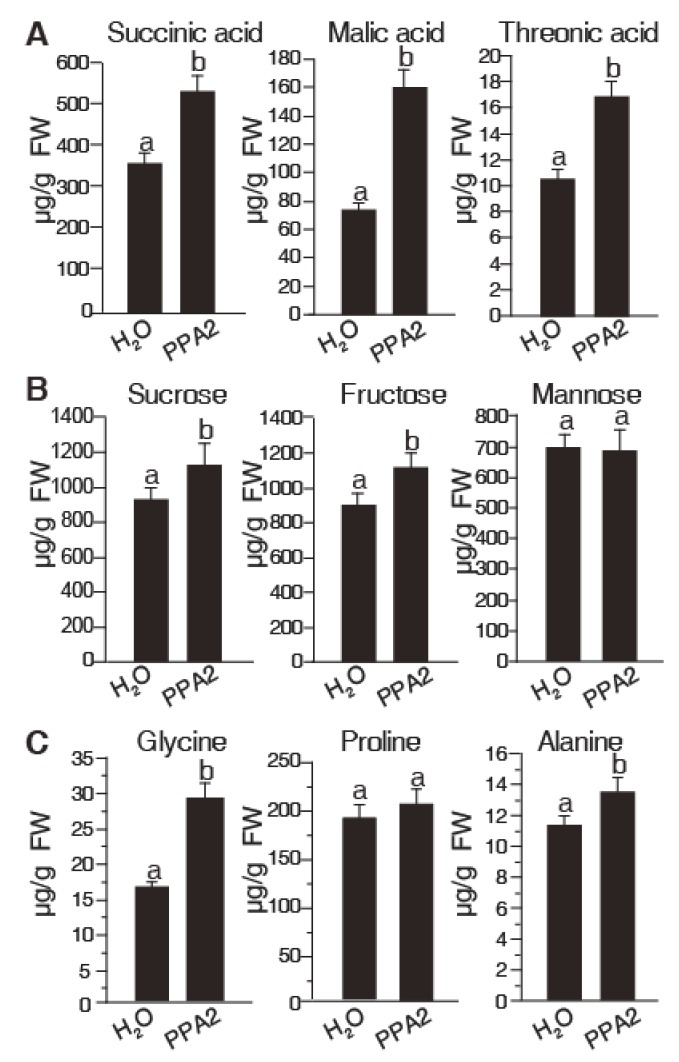
Influence of PPA2 on primary metabolites in rice. Rice seedlings were grown on 0.15% agar medium with or without 10 μM PPA2 for 14 days. GC-MS was used to detect the content of (**A**) organic acids, (**B**) sugars, and (**C**) amino acids as described in Methods. Data were analyzed by using a post hoc multiple *t*-test. Different letters represent significant differences (*p* < 0.05). The experiment was repeated three times by using independent samples. FW, fresh weight.

Plant metabolites are indispensable for plant growth. To further understand PPA2 characteristics, we tested the contents of several primary metabolites, including amino acids, sugars, and organic acids, by GC-MS after plant activator treatment. We placed sterilized rice seeds on 0.15% agar medium containing different test agents (0.3% acetone, 300 μM BTH, or 10 μM PPA2), and then collected samples for further analysis two weeks later. Intriguingly, the contents of organic acids including succinic acid, malic acid, and threonic acid rose significantly in the rice treated with 10 μM PPA2 (Figure 5A), whereas all these three organic acids showed a significant reduction in the plants treated with 300 μM BTH compared to the acetone solvent control (Figure S5A).

In addition, we also analyzed certain sugars and the amino acid contents after the activator treatment. As shown in Figure 5B, sucrose and fructose were significantly accumulated in rice after the 10 μM PPA2 treatment. Besides this, the content of proline, glycine, and alanine were also significantly increased in PPA2-treated rice (Figure 5C). In contrast, all tested sugars and amino acid contents decreased significantly after BTH treatment (Figure S5B,C). These results indicated that the novel activator PPA2 may have a different metabolic regulation mechanism that affects its resistance.

## 3. Discussion

In this study, we reported a new pyrimidine-type plant activator PPA2. PPA2 was a water-soluble compound and did not inhibit plant growth or root system development. PPA2 could induce immune responses against bacterial pathogen infection through a mechanism that may be related to the JA pathway. Moreover, PPA2 might be involved in certain types of primary metabolites to regulate not only plant growth but also plant resistance.

Pretreated PPA2 in plants could induce resistance responses against pathogen attacks, especially bacterial infection. We found that PPA2 pretreatment enhanced the ROS level upon attack by *P. syringae* in *Arabidopsis*, similarly to how our previous reports showed that plant cells generated ROS after plant activator PPA pretreatment to suppress the pathogen proliferation [8]. PPA2 pretreatment also enhanced resistance to the bacterial pathogen *Xoo* in rice, accompanied with significant increases in JA-Ile and OPDA contents. JA is involved in the regulation of host immunity in plants, and many recent studies have demonstrated that JA signaling has an important role in rice resistance to bacterial blight caused by *Xoo* [19,20]. We speculate that PPA2 may increase plant resistance through a mechanism related to the ROS and JA signaling pathways. Although we found that PPA2 showed a limited effect on fungal pathogens in both *Arabidopsis* and rice, its resistance to bacteria is expected to have an application in the future.

Previous studies have shown that BTH-treated plants have a dwarfed appearance because of the cost of resistance-related protein synthesis-known as the fitness cost [21]. We also exhibited that BTH reduced the contents of various primary metabolites in rice (including certain amino acids, sugars, and organic acids), thus leading to a stunted plant growth and reduced root length. This effect may, however, provide a disadvantage in agricultural crops. In contrast with BTH, 10 μM PPA2 does not inhibit rice growth. Moreover, 10 μM PPA2 pretreatment promoted the accumulation of organic acids (succinic acid, malic acid, and threonic acid), sugars (fructose and sucrose), and amino acids (glycine and alanine) in rice. We speculate that this is why BTH and PPA2 have different effects on rice growth [22].

It has been widely recognized that primary metabolites play an important role not only in plant growth and reproduction but also in plant defense [23]. For example, sugars no longer only served as substrates for energy provision, but also acted as real signaling entities with hormone-like actions [24]. The uptake of sugar, managed by the regulation of a host sugar transporter, acted on the antibacterial defense in *Arabidopsis* [25]. Similar results have been obtained in studies on organic acid, showing that its application on plants can protect them against the most dangerous pathogens [26]. Recent metabolite analyses reveal that the levels of several amino acids significantly increase in *Arabidopsis* leaves inoculated with *P. syringae* bacteria [27]. This might explain why PPA2 could help plants resist bacteria rather than fungi pathogen infection. Taken together, our results suggest that PPA2 may have broad potential applications in agriculture against bacterial pathogen attacks.

## 4. Materials and Methods

### 4.1. Materials

*Arabidopsis thaliana* (Col-0) were grown on the soil in the greenhouse at 22 °C with a 16 h light/8 h dark cycle and about 50–60% relative humidity, as described previously [28]. Rice (*Oryza sativa* ssp. *japonica* cv. Nipponbare) seeds were soaked in water (28 °C) for 2–3 days and then sown in soil. The seedlings were grown in a greenhouse under conditions of 16 h light/8 h dark, with a day temperature of 28 °C and 60–80% relative humidity [28]. Benzothiadiazole *S*-methyl ester (BTH) and 6-(methoxymethyl)-2-[5-(trifluoromethyl)-2-pyridyl] pyrimidin-4-ol (PPA2) were purchased from WAKO (Japan) and Maybridge (United Kingdom), respectively. Trypan blue and diaminobenzidine tetrahydrochloride (DAB) were purchased from Sigma.

### 4.2. Pathogen Inoculation

For the bacterial infection, leaves from three-week-old *Arabidopsis* plants were injected either with the virulent *Pseudomonas syringae* pv. *maculicola* (strain DG3) or with 10 mM MgSO_4_ as a mock-inoculation control. Leaf discs were harvested for bacterial quantification at the same times after inoculation, as indicated in previous reports [29]. For the fungus infection, three-week-old *Arabidopsis* wild type plants were sprayed with *Botrytis cinerea* conidia (1 × 10^7^ spores/mL with 2% glucose). After inoculation, plants were covered with a transparent plastic lid to maintain a high humidity and kept in the growth chamber for 24 h in the dark to promote spore germination [28].

To evaluate bacterial blight disease resistance, rice plants were inoculated with *Xanthomonas oryzae* pv. *oryzae* (GDIV strain; abbreviated as *Xoo*) at the five-six-leaf stage by the leaf-clipping method. In brief, the tips of fully stretched rice leaves were cut with scissors dipped in *Xoo* solution (OD_600_ = 0.2–0.5). The disease was scored by measuring the lesion length, and a total of 100 seedlings from each treatment were evaluated 1−2 weeks after inoculation [30].

For the *Magnaporthe grisea* inoculation, rice plants were sprayed with a suspension of conidia (5 × 10^5^ conidia /mL) at the five-six-leaf stage. The blast disease incidence of each replicate (containing 20 seedlings) and a total of 100 seedlings in five replicates of each treatment were evaluated seven days after inoculation. The disease incidence was recorded as the percentage of infected leaves out of all leaves, which was calculated according to the equation: Disease incidence (%) = (number of diseased leaves/total number of leaves assessed) ×100. The disease severity in rice leaves was scored with a rating, denoting the proportions of blast lesions infecting the area over the whole leaf area [31].

### 4.3. DAB and Trypan Blue Staining

Leaves were collected and boiled in lactophenol solution (1:1:1:1 lactic acid:glycerol:liquid phenol:distilled water, *v/v/v/v*) containing 0.025% trypan blue for 30 sec, and then boiled in 2:1 95% ethanol:lactophenol (*v/v*) for 1 min. Leaves were transferred to 50% ethanol for washing and kept in distilled water at 4 °C. The images were photographed under a microscope (Axio Imager A1, Carl Zeiss). For DAB staining, leaves were collected and immersed in 1 mg/mL DAB solution (pH = 5.5) for 2 h, and then boiled in 95% ethanol for 2 min. The leaves were washed in 50% ethanol and then kept in distilled water at 4 °C. Photographs were taken with a stereomicroscope (SteREO Lumar.V12, Carl Zeiss), as described previously [8].

Trypan blue staining was quantified with the cell death index. A scale of 0−2 was used to classify plants according to the percentage of plant tissue affected by cell death (0 = healthy plant or plant without cell death, 1 = plant affected by 1–50%, 2 = 51–100%). The cell death index was calculated according to [32] and described as follows: cell death index=[(0n_o_+1n_1_+2n_2_)/2n] × 100, where n_o_-n_2_ were the numbers of plants with each of the corresponding cell death ratings, and n was the total number of plants assessed (*n* ≥ 100). The final measurement used to quantify the DAB staining was the stained area divided by the total area of the leaf measured using Image J. At least six leaves per treatment were quantified [8]. Trypan blue staining and DAB staining were repeated using three independent samples respectively. Error bars represent ±SE, different letters indicate significant differences based on the PLSD-test (*p* < 0.05).

### 4.4. Effect of Plant Activator on Spore Germination

Slides were coated with 1% agar prepared with or without 10 μM PPA2 by dipping the slides into the agar and taking them out quickly before the agar solidified. The conidia from the *B. cinerea* strain NJ-09 (1 × 10^7^ spores/mL with 2% glucose) were uniformly sprayed on the slides. Slides were placed in a pre-wetted dish and kept in the growth chamber for 12 h in the dark. Spore germination was observed under the microscope, and the germination rate was calculated.

### 4.5. In Vitro Antibacterial Bioassay

The in vitro antibacterial activities of PPA2 were tested against *Xoo* using the turbidimetric assay [33]. The commercially available bactericide bismerthiazol (BMT) was employed as the positive control agent. About 40 μL of solvent NB (3 g of beef extract, 5 g of peptone, 1 g of yeast powder, 10 g of glucose, 1 L of distilled water, pH  =  7.0−7.2) containing the bacterium *Xoo* incubated on the phase of logarithmic growth, was added to 5 mL of solvent NB containing the test compounds or BMT. The bacterial growth was monitored by measuring the optical density at 600 nm (OD_600_), given by turbidity_corrected values_ = OD_bacterium_ − OD_no bacterium_, *I*  =  (C*tur *− T*tur)*/C*tur*  ×  100%. The C_tur_ represented the corrected turbidity value of the bacterial growth of untreated NB (blank control), and T*tur* represented the corrected turbidity value of the bacterial growth of compound-treated NB. The *I* represented the inhibition ratio of the tested compound against the bacterium.

### 4.6. Extraction of Plant Hormones

The hormone extraction was carried out following the method described in [34], with minor modifications. Briefly, the leaf tissue was ground into fine powder with a mortar and pestle in liquid nitrogen, 50 mg of the ground sample was transferred to 2 mL tubes, and 50 μL of internal standards (10 ng d_4_-SA[OlChemim] and 10 ng H_2_-JA[Cerilliant]) was added to each tube. Then, 500 μL extraction buffer (2:1:0.002 2-propanol/H_2_O/HCl, *v/v/v*) was added in each tube. The tubes were kept in a shaker for 30 min at 4 °C. This step was repeated after adding 1 mL dichloromethane to each sample. The sample was then centrifuged at 13,000× *g* for 5 min at 4 °C, and the solvent from the lower phase was transferred into a 2 mL tube and concentrated in a nitrogen evaporator with nitrogen flow. The samples were re-dissolved in 60% methanol and then transferred into glass vials suitable for LC-MS analysis through a Shimadzu UFLC-XR coupled with a hybrid quadrupole time-of-flight mass spectrometer (AB SCIEX Triple TOF 5600+) using a Kinetex C18 column (150 mm × 2.1 mm, 2.6 μm).

### 4.7. Extraction of Metabolites

Metabolites were extracted as described in [35], with modifications. Briefly, the tissues were ground in a mortar and pestle with liquid nitrogen. Next, 50 mg of the ground sample was transferred to 2 mL tubes. Next, 1.4 mL pre-chilled methanol was added, and the mixture was vortexed for 10 s. Then 60 μL of ribitol (0.2 mg/mL stock in dH_2_O) was added as an internal quantitative standard, vortexed for 10 s, and shaken for 10 min at 70 °C. The mixture was centrifuged at 11,000× *g* for 10 min, and the supernatant was transferred to a 10 mL tube. Then, 750 μL pre-cooled chloroform and 1500 μL pre-cooled dH_2_O were added. The sample was vortexed for 10 s and centrifuged for 15 min at 2200× *g*. The supernatant solution was transferred into a fresh tube and then dried in a vacuum concentrator without heating.

For derivatization, 40 μL of methoxyamination reagent was added to each aliquot, and the tubes were shaken for 2 h at 37 °C. Then, 70 μL methoxyamine hydrochloride (20 mg/mL stock in pyridine) was added to the sample aliquots, and the tubes were shaken for 30 min at 37 °C and subsequently transferred into glass vials suitable for GC-MS analysis through an Agilent 7890 A coupled with Agilent 5975C MSD system using an Agilent DB-5MS column (30 m × 0.25 mm, 0.25 μm).

## Supplementary Materials

The following are available online at https://www.mdpi.com/1422-0067/21/8/2705/s1, Figure S1. The impact of PPA2 on fungal pathogen spore germination Figure S2. Pretreatment with PPA2 does not alter plant resistance to *Botrytis cinereal;* Figure S3. PPA2 had no antibacterial activity in vitro*;* Figure S4. PPA2 did not enhance rice resistance to *Magnaporthe grisea*; Figure S5. Influence of BTH on primary metabolites in rice.
