# Supplementary material for: A Pyrimidin-Like Plant Activator Stimulates Plant Disease Resistance and Promotes the Synthesis of Primary Metabolites
Source: Int J Mol Sci. 2020 Apr 14;21(8):2705. doi: 10.3390/ijms21082705 (PMC7215783; doi:10.3390/ijms21082705)
Supplement: Supplementary file 1 [file ijms-21-02705-s001.pdf]

**Supplemental Figure**

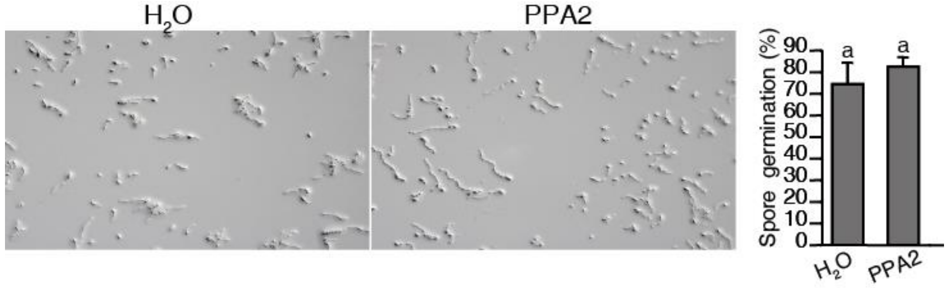


**Supplemental Figure 1. The impact of PPA2 on fungal pathogen spore germination**

The *Botrytis cinerea* spores ($1\times10^7$) were germinated on glass slides covered with 1% agar containing 35 µM PPA2. The spore germination was calculated at 12 h after treatments. Value are means ± SE of at least two independent replicates.

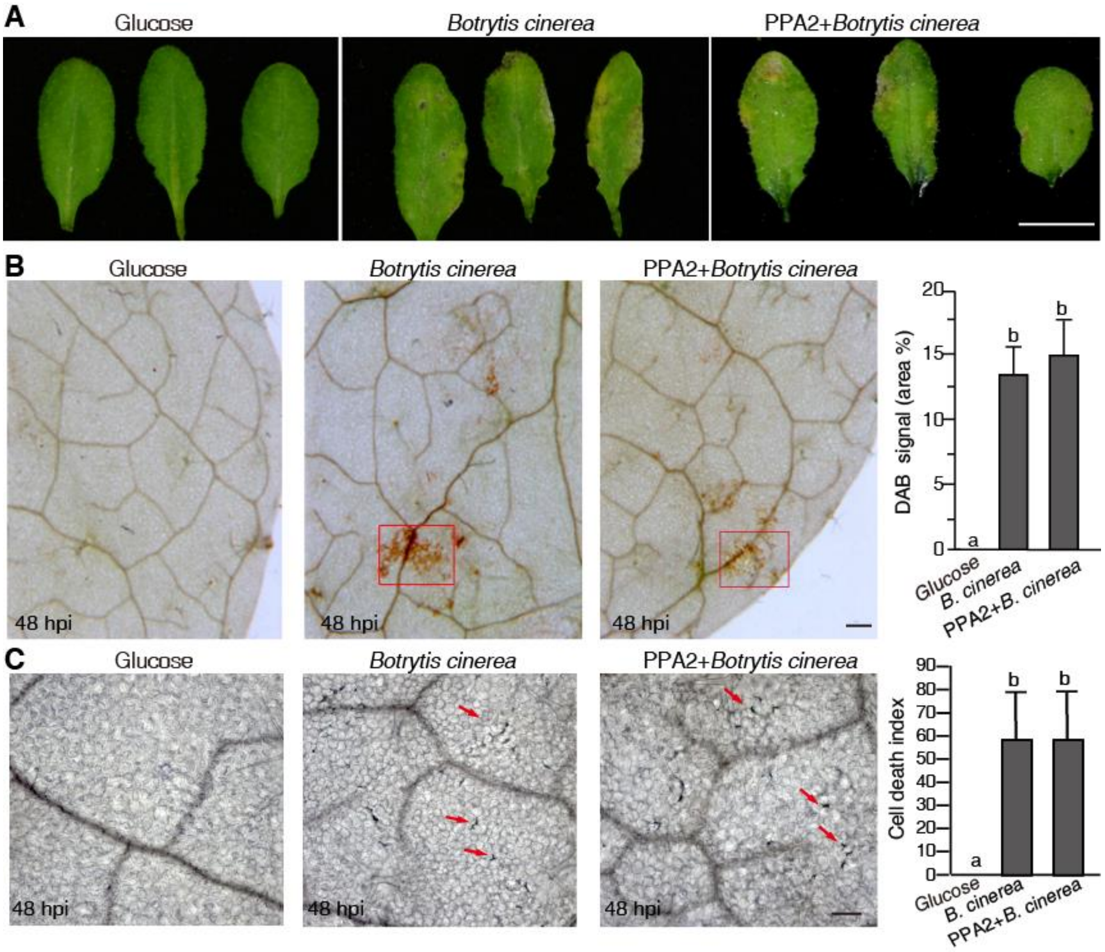


**Supplemental Figure 2. Pretreatment with PPA2 does not alter plant resistance to *Botrytis cinerea***

(**A**) Phenotypes of plant leaves after *B. cinerea* treatments. Three-week-old WT plants were pretreated with $H_2O$ (solvent of PPA2) or 35 μM PPA2 for 3 days, then inoculated with *B. cinerea*. Representative leaves were monitored at 5 dpi with *B. cinerea*. Bar = 20 mm

(**B**) Detection of $H_2O_2$ levels in plant leaves. Three-week-old leaves were pretreated with $H_2O$ (solvent of PPA2) or 35 μM PPA2 for 3 days, then sprayed with *B. cinerea* and assessed 48 h later. DAB staining was conducted as described in Methods. Note brown deposits at the sites of hydrogen peroxide accumulation in plants (square). The right panel showed quantification of DAB staining in each group. Values are means ± SE of at least three independent replicates. Scale bar = 200 μm.

(**C**) Micrograph of trypan blue staining. Three-week-old leaves pretreated with $H_2O$ or 35 μM PPA2 for 3 days, then sprayed with *B. cinerea* and then assessed 48 h later. Note dead cells in leaves (red arrows). The right panel showed quantification of trypan blue staining in each group. Values are means ± SE of at least three independent replicates. Scale bar = 200 μm.

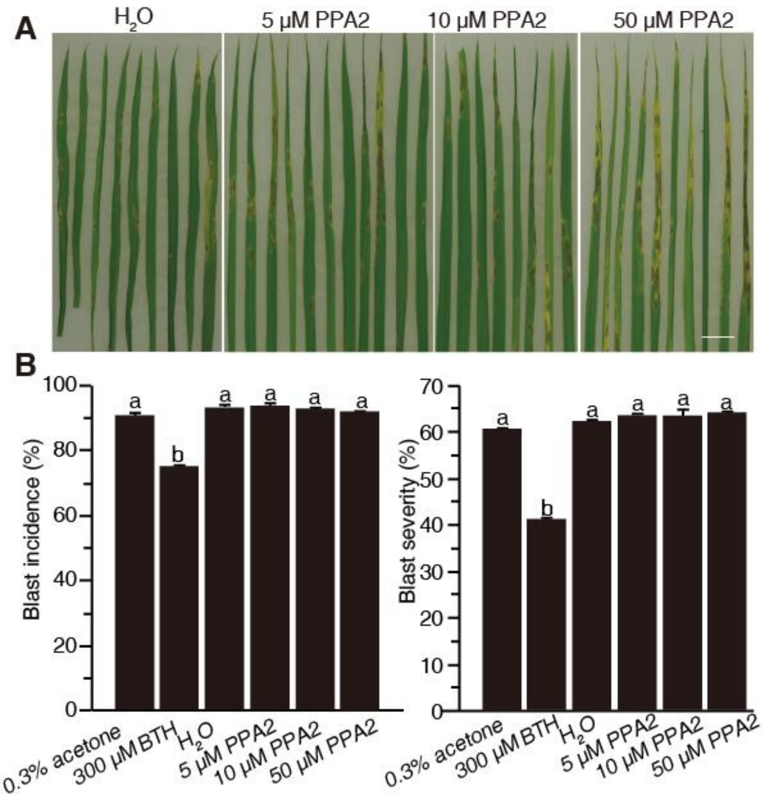


**Supplemental Figure 3. PPA2 did not enhance rice resistance to *Magnaporthe grisea***

Five-six-leaf stage rice seedlings were sprayed with different concentrations of PPA2 for 3 days and then inoculated with *M. grisea*.

(**A**) Phenotypes observation at 7 dpi. Scale bar = 1 cm.

(**B**) Disease incidence and severity. This experiment was repeated twice using independent samples. Different letters above bars represented significant differences using a post hoc multiple *t*-test ($p < 0.05$).

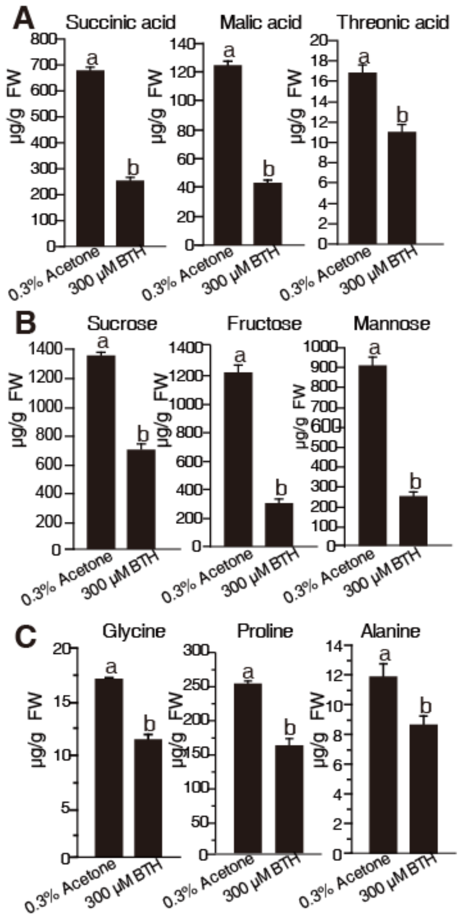


**Supplemental Figure 4. Influence of PPA2 on primary metabolites in rice.**
Rice seedlings were grown on 0.15% agar medium with 0.3% acetone or 300 μM BTH for 14 days. GC-MS was used to detect the content of primary metabolites as described in Methods. Data were analyzed by using a post hoc multiple *t*-test. Different letters represent significant differences ($p < 0.05$). The experiment was repeated three times by using independent samples. FW, fresh weight.
(**A**) Detection the level of organic acids (succinic acid, malic acid and threonic acid) in rice plants treated with or without BTH.
(**B**) Detection the level of sugars (sucrose, fructose and mannose) in rice plants treated with or without BTH.
(**C**) Detection the level of amino acids (glycine, proline and alanine) in rice plants treated with or without BTH.
